# Predicting essential proteins based on subcellular localization, orthology and PPI networks

**DOI:** 10.1186/s12859-016-1115-5

**Published:** 2016-08-31

**Authors:** Gaoshi Li, Min Li, Jianxin Wang, Jingli Wu, Fang-Xiang Wu, Yi Pan

**Affiliations:** 1 School of Information Science and Engineering, Central South University, Changsha, 410083 Hunan People’s Republic of China; 2 Guangxi Key Lab of Multi-source Information Mining and Security, Guangxi Normal University, Guilin, 541004 Guangxi People’s Republic of China; 3 Department of Mechanical Engineering and Division of Biomedical Engineering, University of Saskatchewan, Saskatoon, S7N 5A9 SK Canada; 4 Department of Computer Science, Georgia State University, Atlanta, 30302-4110 GA USA

**Keywords:** Essential proteins, Protein-protein interaction network, Subcellular localization, Orthology

## Abstract

**Background:**

Essential proteins play an indispensable role in the cellular survival and development. There have been a series of biological experimental methods for finding essential proteins; however they are time-consuming, expensive and inefficient. In order to overcome the shortcomings of biological experimental methods, many computational methods have been proposed to predict essential proteins. The computational methods can be roughly divided into two categories, the topology-based methods and the sequence-based ones. The former use the topological features of protein-protein interaction (PPI) networks while the latter use the sequence features of proteins to predict essential proteins. Nevertheless, it is still challenging to improve the prediction accuracy of the computational methods.

**Results:**

Comparing with nonessential proteins, essential proteins appear more frequently in certain subcellular locations and their evolution more conservative. By integrating the information of *s*ubcellular localization, *o*rthologous proteins and PPI *n*etworks, we propose a novel essential protein prediction method, named SON, in this study. The experimental results on S.cerevisiae data show that the prediction accuracy of SON clearly exceeds that of nine competing methods: DC, BC, IC, CC, SC, EC, NC, PeC and ION.

**Conclusions:**

We demonstrate that, by integrating the information of subcellular localization, orthologous proteins with PPI networks, the accuracy of predicting essential proteins can be improved. Our proposed method SON is effective for predicting essential proteins.

## Background

Essential proteins are indispensable in cellular life because even if only one of these proteins is missing, organisms cannot survive or develop. The identification of essential proteins has great significance in the following facts: 1) it helps understand the minimum requirements of the survival and development of a cell. By knowing the minimum requirements of the survival and development of the cell, researchers are able to create a new cell with a minimal genome [1], which is an important content in the emerging synthetic biology. 2) It helps identify disease genes and find novel treatments for diseases [2–4]. Hence, the discovery of essential proteins facilitates to study disease genes. Because essential proteins are indispensable in bacterial cells, they are also the candidates for new antibiotics drug targets.

There are several representative biological methods to identify essential proteins, such as single gene knockout [5], conditional knockout [6] and RNA interference [7]. Because these biological experiment discovery methods are time-consuming, expensive and inefficient, it is appealing to develop novel computational methods to improve the effectiveness of the identification.

Currently, a number of computational identification methods have been proposed. According to the features of essential proteins, these methods can be roughly divided into topology-based methods and sequence-based methods. The topology-based methods are designed based on associations between the essentiality and the topological features of essential proteins in bio-molecular networks. Degree Centrality (DC) [8], Betweenness Centrality (BC) [9], Closeness Centrality (CC) [10], Subgragh Centrality (SC) [11], Eigenvector Centrality (EC) [12], Information Centrality (IC) [13] and Neighborhood Centrality (NC) [14] are the representatives of topology-based methods. CytoNCA [15] is a cytoscape plugin for centrality analysis and evaluation of biological networks, and ClusterViz [16] is a cytoscape APP for cluster analysis of biological network. Additionally, LAC [17], TP and TP-NC [18] are also common topology-based methods.

The topology-based methods are consist of several steps as follows: Firstly, constructing a PPI network G (V, E) based on the pairs of PPI, where V denotes a set of nodes (proteins), and E denotes a set of edges of PPI network. Secondly, constructing an adjacency matrix A of PPI network G, whose element A_u,v_ is 1 if there is an edge between nodes u and v, and 0 otherwise. Then, each protein in PPI network G is scored by using different centrality methods. Finally, essential proteins are determined by their scores.

The key advantage of the topology-based methods is able directly to predict essential proteins without knowing additional information. However, these methods have three main disadvantages as follows: 1) due to a lot of false positives and false negative data in PPI networks, their identification accuracies are affected. 2) These methods have difficulty in predicting essential proteins with low connectivity. 3) These methods ignore the intrinsic biological significance of essential proteins.

The sequence-based methods are another kind of computational methods to predict essential proteins. The sequence-based features are intrinsic features of an individual protein that are determined by genomic sequences. These features have been used by some methods, such as subcellular localization [19], evolutionary conservation [20–22], gene expression [23, 24].

Subcellular localization is an important feature of essential proteins. It represents a concrete location in cells that a certain protein appears. Statistical results show that essential proteins appear more frequently in certain subcellular location than nonessential proteins. Hence, we designed and used protein subcellular localization score based on the features of subcellular localization of proteins.

Evolutionary conservation is also an important characteristic of essential proteins. Because basic life process of a cell is more relevant with essential proteins. The effect of essential proteins in a negative selection is stricter than nonessential proteins [21]. Experimental results have proved that essential proteins evolve more conservative than nonessential proteins.

Gene expression is another important feature of essential proteins. The expression level of mRNA is closely associated with its essentiality. In bacteria, the higher expression level, the slower evolution of protein sequence is [23, 24]. Some studies have shown that protein sequence diversity and protein essentiality are relevant to expression level [25] in eukaryotes. So we draw a conclusion that the expression level of essential genes is higher than that of nonessential ones.

In order to achieve higher identification accuracy, more and more researchers are combining above-mentioned two kinds of methods. By integrating the information of GO annotations with proteins, Li et al. [26] built a weighted PPI network. In addition, by integrating the information of network topology with gene expression, they proposed a centrality method PeC [27]. Based on prior knowledge, network topology and gene expression, they also proposed two new essential protein discovery methods CPPK and CEPPK [28]. Besides the above methods, some researchers proposed to construct dynamic PPI network to reduce the impact of false positives in PPI data [29–31]. Xiao et al. [31] constructed an active PPI network and applied six typical centrality measures to identify essential proteins from the constructed active PPI network. By using PPI network and protein complexes information, Ren et al. [32] proposed an essential protein discovery method named HC. Li et al. [33] proposed a united complex centrality named UC and a parameter controlled method UC-P by using predicting protein complexes [34]. Peng et al. [35] proposed an essential protein discovery method by integrating protein domains and PPI networks. Tang et al. [36] proposed a novel method based on weighted degree centrality by integrating gene expression profiles.

There is other biological information which also was integrated with PPI network to predict essential proteins. Based on random walk model, ION [37] integrates the information of orthologous proteins with PPI networks. Zhao et al. [38] proposed their new method by using overlapping essential modules [39]. Zhong et al. [40] proposed a feature selection method by considering 26 topological or biological features for predicting essential proteins.

In this study, we propose a novel method to predict essential proteins by integrating *s*ubcellular localization, *o*rthology with PPI *n*etwork, named SON.

## Experimental data

This experiment uses multiple datasets, including PPI network dataset, essential protein dataset, subcellular localization dataset and orthologous protein dataset. In order to unify the serial number of proteins in above-mentioned databases, we use the UNIPROT [41] data files to convert protein number in each database.

PPI network dataset of S.cerevisiae is downloaded from DIP database [42] updated to Oct.10, 2010. There are 5093 proteins and 24,743 interactions without self-interactions and repeated interactions in this dataset. We select S.cerevisiae because its PPI data and gene essentiality data are most complete and reliable among various species.

Essential protein dataset is selected from MIPS [43],SGD [44],DEG [45] and SGDP [46]. There are 1285 essential proteins in this dataset, out of which 1167 are in PPI network. We take the 1167 proteins as essential proteins while other 3926(=5093−1167) proteins as nonessential ones.

Subcellular localization dataset of yeast is downloaded from knowledge channel of COMPARTMENTS database [47] on August 30, 2014. It integrates several source databases (UniProtKB [48], MGD [49], SGD [50], FlyBase [51] and WormBase [52]). As a result, it contains 5095 yeast proteins and 206,831 subcellular localization records. We select this database because both its data volume is large and it is updated in a timely manner. After preprocessing, there are still 3923 proteins in PPI network which have subcellular localization information.

Orthologous proteins dataset is taken from Version 7 of InParanoid [53]. It contains a set of pairwise comparisons among 100 whole genomes (1 prokaryote and 99 eukaryotes) that are constructed by INPARANIOD program. We only select the proteins in seed orthologous sequence pairs of each cluster generated by INPARANIOD as orthologous proteins, as it has the best match between two organisms and stands for the high homology.

### Correlation analyses of subcellular localization, orthology and essentiality of proteins

To understand associations between subcellular localization and essentiality of proteins, we first count the number of essential and nonessential proteins in each subcellular location, respectively. Next, their ratios are calculated. The results are shown in Table 1. According to Table 1, the ratios of essential proteins are higher than that of nonessential proteins in Cytoskeleton, Golgi apparatus, Cytosol, Nucleus and Endoplasmic reticulum. Hence, the five subcellular locations above mentioned are positively correlated with essential proteins while the others are negatively correlated.

**Table 1:** Number and ratio of essential and nonessential proteins in each subcellular location

Subcellular location	Essential proteins number	Essential proteins ratio	Nonessential proteins number	Nonessential proteins ratio
Cytoskeleton	95	0.081	133	0.033
Golgi apparatus	61	0.052	184	0.046
Cytosol	138	0.118	289	0.073
Endosome	22	0.019	109	0.027
Mitochondrion	173	0.148	753	0.189
Plasma membrane	53	0.045	354	0.089
Nucleus	809	0.693	1407	0.353
Extracellular space	1	0.001	70	0.018
Vacuole	19	0.016	238	0.060
Endoplasmic reticulum	137	0.117	292	0.073
Peroxisome	4	0.003	61	0.015

To understand the association between subcellular localization and essentiality of proteins, we first count the number of essential and nonessential proteins in each subcellular location, respectively. Next, their ratios are calculated. According to Table 1, the ratios of essential proteins are higher than that of nonessential proteins in Cytoskeleton, Golgi apparatus, Cytosol, Nucleus and Endoplasmic reticulum. Hence, the five subcellular locations above mentioned are positive correlation with essential proteins while the others are negative correlation

The associations between orthology and essentiality of proteins have been verified by Peng et al. [37]. The ratio of essential proteins is 51 % if the proteins have orthologs for at least 80 species. But if the proteins have no orthologs for reference organisms, the ratio of essential proteins is about 22 %, near to random probability [54].

## Methods

Our novel method, SON, predicts essential proteins based on the information integration of subcellular localization, Orthology and PPI network. In the following subsections, we will introduce how to use these information and integrate them to calculate a protein’s essentiality.

### Network Centrality based on edge clustering coefficient (NC)

In the previous studies, it has been shown that network centrality is an important measure for predicting essential proteins and the network centrality based on edge clustering coefficient [14] is one of the most effective measures for the identification of essential proteins. Given a PPI network *G =* (*V, E*) and a protein *i*, its network centrality based on edge clustering coefficient *NC*(*i*) is defined as the sum of edge clustering coefficients of all edges directly connected with protein *i* in the graph *G*.

$$ NC(i)={\displaystyle \sum_{j\in {N}_i}}ECC\left(i,j\right) $$

1 $$ ={\displaystyle {\sum}_{j\in {N}_i}}\frac{Z_{i,j}}{min{\left({k}_i-1,{k}_j-1\right)}_{\boxed{}}} $$

where *N*_*i*_ denotes the set of all neighbors of protein *i, Z*_*i, j*_ is the number of triangles built on edge(*i,j*), *k*_*i*_ and *k*_*j*_ are the degrees of nodes *i* and *j*, respectively. *min*(*k*_*i*_*−1*, *k*_*j*_*−1*) represents the maximal possible number of triangles that might potentially include the edge(*i,j*).

The edge clustering coefficient (ECC) is used to measure the degree of closeness between two nodes in a graph which has been widely applied in identifying network modules [55, 56]. Those edges which have higher ECC value are more likely to be in a module. It has been shown that essential proteins and disease genes tend to appear in the same cluster [57–59]. Therefore, if an edge with high ECC value, it is more likely to be a connection of two essential proteins. Obviously, a protein which has more neighbors and gets higher ECC values with its neighbors will have a relatively higher NC value and will tend to be an essential protein. In order to match with orthologous score and subcellular localization score whose value ranges are [0,1], here we use the normalized NC value for each protein, denoted as NNC. For a protein *i*, its normalized NC value *NNC*(*i*) is defined as:

2 $$ NNC(i)=NC(i)/ Max\_NC $$

where *Max_NC* denotes the maximum *NC* value of all the proteins in the graph *G*.

### Subcellular localization score

It has been shown that proteins must be localized at their appropriate subcellular compartments to perform their desired functions and thus the subcellular localization information is helpful to the identification of essential proteins [59]. Here, we analyzed the associations between the subcellular localization and the topology of PPI networks. All the proteins in the PPI network are sorted in descending order according to their NNC scores. Then we calculate the numbers of subcellular location *l* where the top *k*% proteins appear and where the bottom *k*% proteins appear, respectively. Considering that more counting proteins may result in more false positives, we use *k* = 5 in this paper, ie., that the top/bottom 5 % proteins are selected. Let *f*_*l*_ be the frequency of *l* where the top *k*% proteins appear and *h*_*l*_ denote the frequency of *l* where the bottom *k*% proteins appear. Subcellular Localization Correlation Coefficient *LCC(l)* is defined as

3 $$ LCC(l)=\left\{\begin{array}{c}\hfill 1-\frac{h_l}{f_l},\kern0.5em \left|{f}_l<{h}_l\right.\hfill \\ {}\hfill \frac{f_l}{h_l}-1,\kern0.5em {f}_l\ge {h}_l\hfill \end{array}\right.\begin{array}{c}\hfill \boxed{}\hfill \\ {}\hfill \boxed{}\hfill \end{array} $$

When *f*_*l*_ < *h*_*l*_, more proteins with low NNC values appear in the location *l* and a negative relationship is thought to be between the location *l* and protein’s essentiality. On the contrary, there is a positive correlation between the location *l* and protein’s essentiality when *f*_*l*_ ≥ *h*_*l*_. When *f*_*l*_ = 0, we set *LCC*(*l*) as the maximum of $1-\frac{h_l}{f_l}$ with *f*_*l*_ ≠ 0. When *h*_*l*_ = 0, we set *LCC*(*l*) as the maximum of $\frac{\ {f}_l}{\ {h}_l}-1$ with *h*_*l*_ ≠ 0. A protein may appear in multiple subcellular locations. For a protein *i*, its subcellular localization score *SL*(*i*) is defined as the sum of *LCC*(*l*) of all the subcellular locations it appears. Here, for each protein *i* we also use the normalized *SL* value *NSL*(*i*) by using the following formula:

4 $$ NSL(i)=\frac{SL(i)+ Max\_\left|SL\right|}{Max\left(SL(i)+ Max\_\left|SL\right|\right)} $$

Where *Max*_|*SL*| denotes the maximum value of |*SL*(*i*)| for all the proteins in *G* and Max in the denominator takes for all the proteins in *G*.

### Orthologous score

Orthologous score method of SON comes from ION method [37]. Given a PPI network *G = (V, E)*, let *S* be the set of reference species which is used to get orthologous information of *V. s* denotes its element and |*S*| denotes the number of its elements. Let *X*_*s*_ be a subset of *V* whose element has orthologs in organism *s*. For a protein *i,* its orthologous score *OS*(*i*) is defined as the number of reference organisms in which the protein *i* has orthologs, where *i* ∈ *V* (*i* = 1, …, *N*)*.* Similar to the network centrality based on edge clustering coefficient and subcellular localization score, we also use the normalized *OS* value *NOS*(*i*) by using the following formula:

5 $$ NOS(i)=\frac{OS(i)}{Max\_ OS} $$

Where *Max*_*OS* denotes the maximum value of *OS(i)* for all the proteins in G.

According to the above definition, a protein’s orthologous score is 1 if its orthologs in all organisms included in set *S*. On the contrary, its orthologous score is 0 if it does not have orthologs in any organisms in set *S*.

### The sorting score and SON algorithm

The sorting score of our algorithm SON is a linear combination of the three scores: normalized network centrality based on edge clustering coefficient *NNC*(*i*), normalized subcellular localization score *NSL*(*i*), and normalized orthologous score *NOS*(*i*). For a protein *i*, its sorting score is calculated as follows:

6 $$ pr(i)=\left(1-\alpha \right)*NOS(i)+\alpha \left[\left(1-\beta \right)*NSL(i)+\beta *NNC(i)\right] $$

where *α* ∈ [0, 1] and *β* ∈ [0, 1] are used to adjust the proportion of these three scores.

SON algorithm is introduced as follows.

- SON algorithm
  - Input: A PPI network represented as a graph *G = (V, E)*, the scoring table of subcellular localization of proteins, orthologs datasets between Yeast and 99 other organisms, parameter *α*, parameter *β*.
  - Output: Top *K* percent of proteins sorted by *pr* in descending order.
    - Step1: Calculate the value of NNC for each protein by using Equation (2).
    - Step2: Calculate the score of subcellular localization for each protein by using Equation (4).
    - Step3: Calculate orthologous score for each protein by using Equation (5).
    - Step4: Calculate the value of pr for each protein by using Equation (6).
    - Step 5: Sort proteins by the value of *pr* in descending order.
    - Step 6: Output top *K* percent of sorted proteins.

## Results and discussion

In order to analyze and evaluate the performance of our method, SON, we perform a large number of experiments on these datasets. There are 5093 proteins and 24,743 interactions in PPI network of S.cerevisiae. Essential protein dataset is constructed by integrating MIPS,SGD,DEG and SGDP which has 1167 essential proteins in PPI network. Subcellular localization dataset includes 5095 yeast proteins and 206,831 subcellular localization records. After preprocessing, there are 3923 proteins in this dataset that have subcellular localization records. Orthologous proteins dataset is taken from Version 7 of InParanoid consisting a set of pairwise comparisons between 100 whole genomes.

In this section, we first analyze the influence of two parameters *α* and *β* towards the performance of SON algorithm. Then, SON is compared with the other existing algorithms, such as DC, BC, CC, SC, EC, IC, NC, PeC and ION. We adopted three types of popular comparison methodologies: 1) Histogram comparison methodology. Firstly, the results are sorted in descending order. Next, to select the top 1, 5, 10, 15, 20 and 25 % proteins as candidate essential proteins. Then, we compare prediction results based on the set of known essential proteins. The performance is presented in the form of histograms of the number of essential proteins predicted by each algorithm. 2) Precision-recall curves methodology. 3) Jackknife methodology. In the end, the differences of these algorithms which have high connectivity proteins and low ones are analyzed in detail.

### Influence of parameter *α* and *β*

**Fig. 1:**
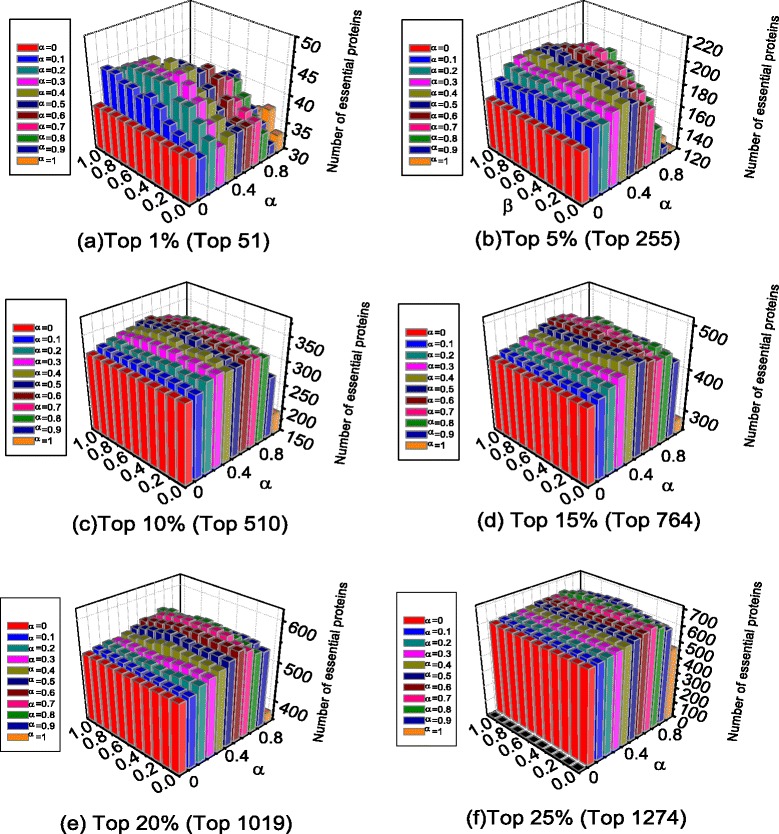
Influence of parameters α and β. (**a**) Top 1 % (Top 51) (**b**) Top 5 % (Top 255) (**c**) Top 10 % (Top 510) (**d**) Top 15 % (Top 764) (**e**) Top 20 % (Top 1019) (**f**) Top 25 % (Top 1274)

In our novel method, SON, the scoring of proteins is associated with parameters *α* and *β*. The value ranges of *α* and *β* are both from 0 to 1. When the values of *α* and *β* take 0, 0.1, 0.2, …, 0.9, 1, respectively, the number of essential proteins predicted by SON are shown in Fig. 1.

As shown in Fig. 1, when *α* values from 0.2 to 0.8 and *β* from 0.3 to 0.7, simultaneously, the result of SON is better. In particular, when *α* = 0, namely only orthologous information is used, parameter *β* has no effect, all the results are the same.

**Fig. 2:**
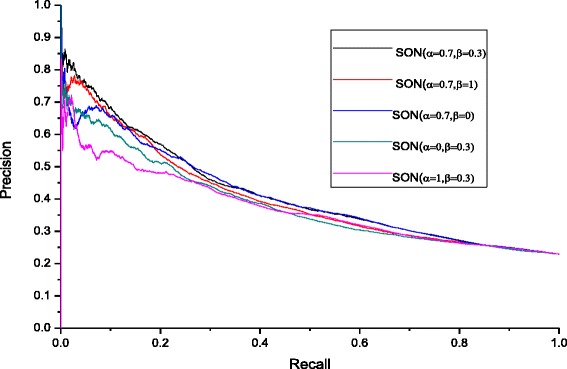
Influence of parameters *α* and *β* for SON

In order to further analyze the influence of the parameters *α* and *β*, we utilize the precision-recall curves methodology with five sets of parameters *α* and *β*, such as *α* = 0.7and *β* = 0.3,*α* = 0.7 and *β* = 1,*α* = 0.7 and *β* = 0,*α* = 0 and *β* = 0.3, *α* = 1 and *β* = 0.3. The results are shown in Fig. 2. According to Fig. 2, when *α* = 0.7and *β* = 0.3, namely, the proportions of orthologous information, NC, and subcellular localization information are 30, 21, and 49 %, respectively, the result is the best. In this paper, we consider the optimal values to be *α* = 0.7 and *β* = 0.3.

### Comparison with nine existing methods

**Fig. 3:**
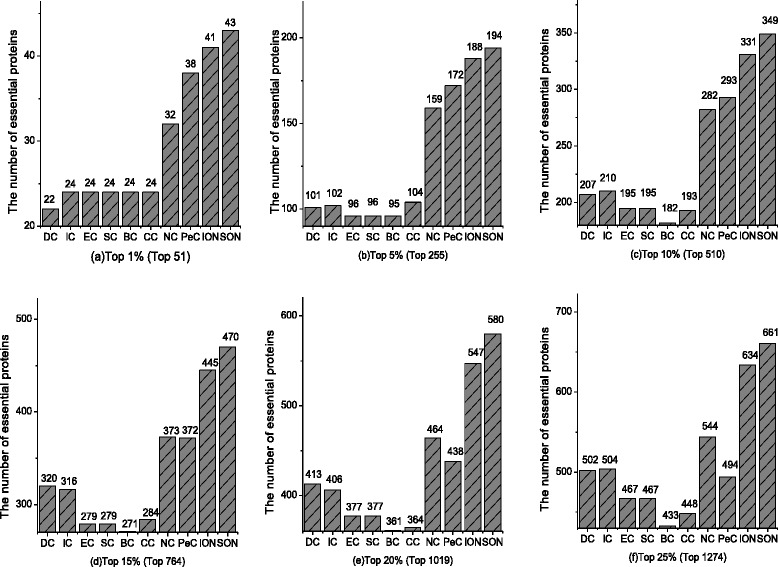
SON compared with several existing methods. (**a**) Top 1 % (Top 51) (**b**) Top 5 % (Top 255) (**c**) Top 10 % (Top 510) (**d**) Top 15 % (Top 764) (**e**) Top 20 % (Top 1019) (**f**) Top 25 % (Top 1274)

In this section, the performance of SON is compared with nine existing methods. We select the top 1, 5, 10, 15, 20 and 25 % proteins predicted by DC, BC, CC, SC, EC, IC, NC, PeC, ION and SON as candidate essential proteins to compare, respectively. The results are shown in Fig. 3. From Fig. 3, it is easy to see that the result of SON is clearly the best.

### Comparison the experimental results based on precision-recall curve

**Fig. 4:**
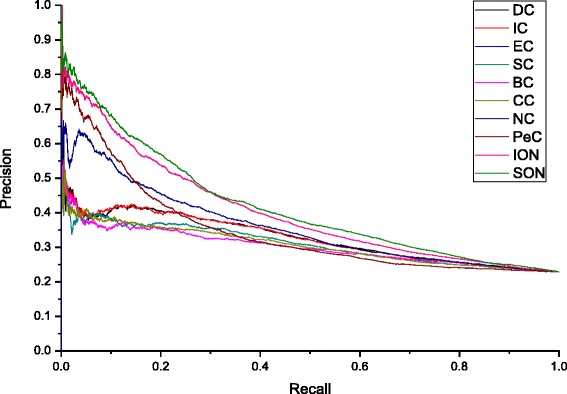
PR curves of SON and that of other methods

Precision-recall (PR) curve is another common methodology to validate algorithm performance. In terms of the corresponding area under the PR curve (AUC) value, the overall performance of each method is evaluated. At the beginning, according to their scores computed for each method, all proteins are sorted in descending order. Then the top *K* proteins are selected as candidate essential proteins while the remaining proteins in PPI networks as candidate nonessential ones. The values of *K* range from 1to 5093. The results are shown in Fig. 4. As shown in Fig. 4, PR curve of SON is obviously higher than that of other methods. Note that the curves of EC and SC are almost identical.

### Comparison the experimental results based on jackknife methodology

**Fig. 5:**
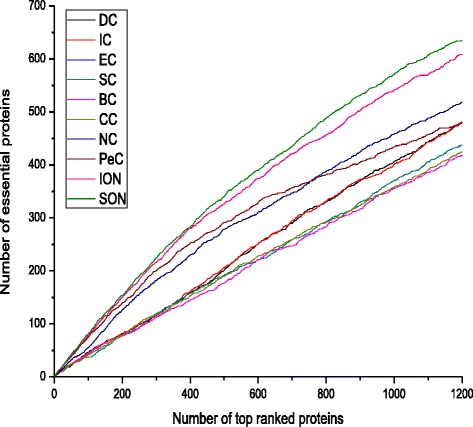
Jackknife curves of SON and other nine methods

To further investigate the performance of SON, jackknife methodology is also employed. The results are shown in Fig. 5. The x-axis represents the number of proteins in PPI networks ranked in descending order according to their sorting scores computed from all above-mentioned methods while the y-axis represents the cumulative count of essential proteins. The areas under the curves are used to measure the performances of the above-mentioned methods. According to Fig. 5, SON is clearly better than DC, IC, EC, SC, BC, CC, NC, PeC and ION. Note that the curves for EC and SC are almost identical.

### Differences between SON and nine existing methods

In order to further analyze SON, we compared its ability to identify low/high connectivity essential proteins with nine existing methods. After statistical analysis, we notice that the low connectivity (less than or equal to 10) proteins are about 76 % in the yeast PPI network and 58 % of essential proteins in known essential protein list are low connectivity in the yeast PPI network. Hence, it is very important for essential protein prediction method to identify low connectivity essential proteins. The results of predicting essential proteins with low and high connectivity for several above-mentioned methods are illustrated in Table 2.

**Table 2:** Number of predicting high and low connectivity essential proteins by using SON and other nine existing methods

	*K*	DC	IC	EC	SC	BC	CC	NC	PeC	ION	SON
degree < =10	1 %	0	0	0	0	0	0	0	0	17	14
	5 %	0	0	0	0	0	0	3	40	66	64
	10 %	0	0	0	0	1	0	27	84	108	116
	15 %	0	0	8	8	18	7	66	117	146	156
	20 %	0	0	28	28	41	20	101	153	188	193
	25 %	11	20	73	73	76	55	156	192	253	220
degree > 10	1 %	22	24	24	24	24	24	32	39	24	29
	5 %	101	102	96	96	95	104	156	133	122	129
	10 %	207	210	195	195	181	193	255	209	223	233
	15 %	320	316	271	271	253	277	307	255	299	313
	20 %	413	406	349	349	320	344	363	285	359	387
	25 %	491	484	394	394	357	393	388	302	381	441

As shown in the top part of Table 2 (degree < = 10), it is weak for eight centrality methods to predict low connectivity essential proteins. When taking the top 20 % proteins ranked in descending order according to their ranking scores computed by DC and IC, the numbers of predicting essential proteins are 0. The performance of SON overall is better than that of eight centrality methods (DC, IC, EC, SC, BC, CC, NC and PeC). When *K* is 10, 15, 20 %, respectively, the performance of SON is also better than that of ION

As shown in the top part of Table 2 (degree ≤ 10), it is weak for eight centrality methods to predict low connectivity essential proteins. When taking the top 20 % proteins from DC and IC, the numbers of predicting essential proteins are 0. The performance of SON overall is better than that of eight centrality methods (DC, IC, EC, SC, BC, CC, NC and PeC). When *K* is 10, 15 and 20 %, respectively, the performance of SON is also better than that of ION.

As shown in the bottom part of Table 2 (degree > 10), we can see that DC and IC have good performance in predicting high connectivity essential proteins. However, SON in predicting high connectivity essential proteins outperforms EC, SC, BC, CC and ION.

## Conclusions

Although identification of essential proteins is of great significance, biological experimental methods for identifying essential proteins are time-consuming, costly and inefficient. Hence it is necessary to use computational methods to identify essential proteins. In this paper, by the integration of subcellular localization, orthologous and PPI, we proposed a novel method, SON, to predict essential proteins.

First, we analyze the correlation between subcellular localization, orthologous proteins and essentiality of proteins. Then, we propose our novel method SON. By comparing with nine existing methods (DC, IC, EC, SC, BC, CC, NC, PeC and ION), we conclude that the overall performance of SON is the best among them. We further analyze the performance of SON in predicting low/high connectivity essential proteins, and discover that SON can predict a large number of low connectivity essential proteins ignored by the eight existing centrality methods.
